# Impairment in inflammasome signaling by the chronic *Pseudomonas aeruginosa* isolates from cystic fibrosis patients results in an increase in inflammatory response

**DOI:** 10.1038/s41419-021-03526-w

**Published:** 2021-03-04

**Authors:** Melissa S. Phuong, Rafael E. Hernandez, Daniel J. Wolter, Lucas R. Hoffman, Subash Sad

**Affiliations:** 1 Department of Biochemistry, Microbiology and Immunology, Faculty of Medicine, University of Ottawa, Ottawa, ON Canada; 2 Center for Global Infectious Diseases Research, Seattle Children’s Research Institute, Seattle, WA USA; 3 Department of Pediatrics, University of Washington, Seattle, WA USA; 4 Department of Microbiology, University of Washington, Seattle, WA USA; 5 University of Ottawa Centre for Infection, Immunity and Inflammation (CI3), Ottawa, ON Canada

**Keywords:** Cell death, Immune cell death

## Abstract

*Pseudomonas aeruginosa* is a common respiratory pathogen in cystic fibrosis (CF) patients which undergoes adaptations during chronic infection towards reduced virulence, which can facilitate bacterial evasion of killing by host cells. However, inflammatory cytokines are often found to be elevated in CF patients, and it is unknown how chronic *P. aeruginosa* infection can be paradoxically associated with both diminished virulence in vitro and increased inflammation and disease progression. Thus, we investigated the relationship between the stimulation of inflammatory cell death pathways by CF *P. aeruginosa* respiratory isolates and the expression of key inflammatory cytokines. We show that early respiratory isolates of *P. aeruginosa* from CF patients potently induce inflammasome signaling, cell death, and expression of IL-1β by macrophages, yet little expression of other inflammatory cytokines (TNF, IL-6 and IL-8). In contrast, chronic *P. aeruginosa* isolates induce relatively poor macrophage inflammasome signaling, cell death, and IL-1β expression but paradoxically excessive production of TNF, IL-6 and IL-8 compared to early *P. aeruginosa* isolates. Using various mutants of *P. aeruginosa*, we show that the premature cell death of macrophages caused by virulent bacteria compromises their ability to express cytokines. Contrary to the belief that chronic *P. aeruginosa* isolates are less pathogenic, we reveal that infections with chronic *P. aeruginosa* isolates result in increased cytokine induction due to their failure to induce immune cell death, which results in a relatively intense inflammation compared with early isolates.

## Introduction

Cystic fibrosis (CF) is a genetic disease caused by a mutation in the cystic fibrosis transmembrane conductance regulator (CFTR). The morbidity and mortality due to CF are attributable to lung disease^1^. Loss of CFTR function results in altered airway surface fluid viscosity and chemistry and impaired secretion clearance by the airways^1^. This renders CF patients vulnerable to opportunistic infections with a variety of microbes, of which *Pseudomonas aeruginosa* (*P. aeruginosa*) is increasingly common as patients age^1^.

Inflammatory cell death is a key mechanism of host defense against pathogens, which results in the lysis of infected phagocytes through pyroptosis and secretion of the IL-1 cytokine family following assembly of intracellular inflammatory platforms called inflammasomes^2^. Among the various inflammasome pathways encoded by phagocytes, the most significant for CF *P. aeruginosa* respiratory infections are the Nod-like receptor protein 3 (NLRP3) and Nod-like receptor containing a CARD domain 4 (NLRC4) pathways^3^.

*P. aeruginosa* undergoes genetic adaptations during chronic infection that allows this pathogen to evade typical immune processes. Many of the downregulated bacterial products are also toxic to host cells in vitro and, in addition to being recognized by the host’s immune system, are important for pathogenesis^4–6^. Therefore, chronic CF infection isolates of *P. aeruginosa* are considered less virulent than early infection isolates. However, chronic infection with *P. aeruginosa* accelerates the decline in lung function, leading to increases in patient morbidity and mortality^7–9^. Antibiotic treatment targeting *P. aeruginosa* results in a reduction in proinflammatory cytokines in CF patients’ bronchoalveolar lavage fluid (BALF), indicating an important role for *P. aeruginosa* in stimulating airway inflammation^10^. This persistent inflammatory response is thought to play a central role in the pathogenesis of CF lung disease^11,12^. Given this apparent paradox, it is unclear if the chronic isolates of *P. aeruginosa* from CF patients are less virulent or if they can still induce proinflammatory mechanisms. In this report, we investigated the mechanism behind this dichotomy. Using *P. aeruginosa* isolates from early and chronic CF respiratory infections and specific transposon insertion mutants, we show that chronic isolates, and strains with the same genetic adaptations as those isolates, induced less cell death yet higher levels of proinflammatory cytokines compared with their early-infection, unadapted counterparts. We reveal that the culling of infected cells is a regulatory mechanism that limits the expression of inflammatory cytokines.

## Materials and methods

### Bacterial sample collection and selection

Clinical isolates of *P. aeruginosa* were collected retrospectively after the isolation and identification of *P. aeruginosa* from routine CF patient respiratory cultures performed in the clinical microbiology laboratories at Seattle Children’s Hospital and the University of Washington Medical Center. Both bacterial isolates and the associated data were coded and de-identified by the CF Isolate Core prior to transfer to the researchers. *P. aeruginosa* isolates from early (*n* = 15) and chronic (*n* = 10) infections from CF patients were obtained from the Seattle CF Isolate Core. Isolates from early infections were identified as the first positive *P. aeruginosa* culture for patients seen at Seattle Children’s whereas isolates from chronic infections were identified in patients whose sputum cultures were positive *P. aeruginosa* for most samples for at least 4 years. A second longitudinal, clonally related cohort of isolates was also obtained from the Seattle CF Isolate Core in which phylogenic relationships were established using previous pulsed-field gel electrophoresis data from the PASA collection of *P. aeruginosa* isolates^13^. For this group, early (*n* = 15) and chronic (*n* = 16) isolates were separated by at least 1.5 years from seven patients.

Environmental and acute, non-CF infection *P. aeruginosa* isolates were previously acquired from Dr. G. Perron (Department of Biology, University of Ottawa) and described previously^14^. Wild-type PA14, PAO1, and *Staphylococcus aureus* subsp. *aureus* Rosenbach (ATCC® 6538™) (*S. aureus* 6538) were used as reference strains. Both PA14 and PAO1 are commonly used reference strains for pathogenesis studies, and PA14 was originally isolated from a burn patient^15^ while PAO1 was isolated from a wound^16^. In addition, transposon mutants of PA14 (PA14 *popB*::tn, *popD*::tn, *fliC*::tn, and *exsA*::tn) were used for in vitro infections and were acquired from the PA14 Transposon Insertion Mutant Library^17^.

### Bacteria growth conditions and infection of cells

All acquired isolates were streaked onto LB agar, grown in LB medium overnight, and diluted and grown in liquid medium to the mid-log phase (final OD_600_ ∼ 0.5) to make new glycerol stocks to be stored at −80 °C. Bacterial isolates were grown to the mid-log phase, and various MOIs were used to infect THP-1 macrophages in triplicates. When exposing cells to heat-killed bacteria, the liquid bacterial culture was boiled at 95 °C for 10 min prior to the preparation of serial dilutions. After centrifugation at 2500 rpm for 7 min, the infections were terminated at 3 h postinfection. For time-course experiments, infections were terminated at 1-h intervals.

### Cell culture

THP-1 monocyte cells (ATCC® TIB-202™) were grown in RPMI 1640 medium supplemented with 8% fetal bovine serum (FBS) and 50 μg/mL of gentamicin. The reporter cell line THP1-Lucia™ NF-κB (InvivoGen) was used for detecting NF-κB activation after exposure to heat-killed bacteria. THP1-defCASP1 (InvivoGen), a cell line deficient in caspase-1, was used to determine caspase-1 involvement during infections. Both cell lines were grown according to recommended conditions. In brief, the cells were cultured in RPMI 1640 medium supplemented with 8% FBS, 25 mM HEPES, 100 µg/mL of Normocin™ (InvivoGen), and 50 μg/mL gentamicin. On alternating passages, 100 µg/mL of Zeocin™ (InvivoGen) was added to the culture medium to maintain selective pressure. To differentiate the THP-1 cells into a macrophage phenotype, 50 ng/mL of phorbol 12-myristate 13-acetate (PMA) was added to the culture medium for 72 h. Afterwards, the cells were washed with phosphate-buffered saline (PBS) and maintained in RPMI 1640 medium supplemented with 8% FBS without antibiotics and PMA for 24 h.

To generate primary human macrophages, peripheral blood mononuclear cells (PBMCs) were isolated from the blood of a healthy donor using SepMate™ tubes (STEMCELL Technologies, 85450) and density gradient medium, Lymphoprep™ (STEMCELL Technologies, 07801) according to manufacturer’s instructions. After washing the PBMCs twice with PBS, 10^7^ cells were added to a polystyrene petri dish (100 mm × 15 mm) coated with 100 μL of 1 μg/mL recombinant human M-CSF (R&D Systems, 216-MC). RPMI supplemented with 10% FBS was added up to 10 mL per dish. Cells were incubated at 37 °C in a 5% CO_2_ incubator for 6 days to allow macrophage differentiation to occur. The entire cell culture was harvested on day 6 and seeded in a 96-well plate at 5 × 10^4^ cells per well.

NuLi-1 (ATCC® CRL-4011™) cells, a human bronchial epithelial (HBE) cell line, were grown in BEGM™ Bronchial Epithelial Cell Growth Medium (Lonza, CC-3171) supplemented with SingleQuots Supplements and Growth Factors™ (Lonza, CC-4175) and 50 μg/mL of gentamicin. Flasks and culture plates used to grow NuLi-1 cells were coated in 50 µg/mL of collagen Type 4 (Sigma, C7521).

### Cell viability, cytokine assays, and western blotting

Cell viability was evaluated by measuring the uptake of neutral red postinfection that were performed in triplicates, as has been described previously^14^. Supernatants were collected at 1–3 h postinfection and cytokines were measured using human IL-1β (DY201), TNF (DY210), IL-8 (DY208), and IL-6 (DY206) (R&D Systems, USA) and IL-10 (555157) (BD Biosciences, USA) ELISA kits according to the manufacturers' instructions. Cell lysates were obtained by using 1% SDS lysis buffer containing 1% 2-ME 1 or 3 h postinfection, boiled for 5 min, and subjected to western blot analysis as published previously^14^.

### Antibodies

The following antibodies were used for western blotting: rabbit anti-phospho-p38 MAPK (Thr180/Tyr182) (D3F9) (#9211; Cell Signaling), rabbit anti-p38 MAPK (#9212; Cell Signaling), rabbit anti-phospho-NF-κB p65 (Ser536) (93H1) (#3033; Cell Signaling), rabbit anti-NF-κB p65 (D14E12) (#8242; Cell Signaling), rabbit anti-phospho-MAPKAPK-2 (Thr334) (27B7) (#3007; Cell Signaling), rabbit anti-MAPKAPK-2 (#3042; Cell Signaling), rabbit anti-active caspase-1 (#4199; Cell Signaling), rabbit anti-caspase-1 (#3866; Cell Signaling), mouse anti-caspase-8 (8CSP03) (sc-56070; Santa Cruz Biotechnology), rabbit anti-caspase-5 (D3G4W) (#46680; Cell Signaling), and mouse anti-β-actin (8H10D10) (#3700; Cell Signaling). All primary antibodies were diluted 1:1000. Secondary antibodies used were goat anti-rabbit IgG, HRP-linked antibody (#7074; Cell Signaling) and horse anti-mouse IgG, HRP-linked antibody (#7076; Cell Signaling), and they were diluted to 1:5000. Blots were developed with an enhanced chemiluminescence substrate (Thermo Scientific) and bands were identified by exposure of the membrane to X-ray film (Kodak, CareStream Health).

### NF-κB detection with reporter cell line

In vitro infections with heat-killed *P. aeruginosa* were conducted with the THP1-Lucia™ NF-κB macrophages seeded in 96-well plates and in triplicates, as previously described for the THP-1 cells. 3 h postinfection, 20 µL of the supernatant from each well was transferred to a white opaque plate (Nalge Nunc International). In each well, 50 µL of QUANTI-Luc™ (InvivoGen) was added, and luminescence was immediately measured using a FilterMax F5 plate reader (Molecular Devices).

### RNA extraction and quantitative real-time PCR (qPCR) analysis

THP-1 macrophages were infected with select *P. aeruginosa* isolates from CF patients for 1 h. RNA extractions were conducted using an RNeasy Mini Kit (Qiagen) as per the manufacturer’s instructions, and RNA was stored in −80 °C prior to use. cDNA was synthesized using the iScript cDNA Synthesis Kit (Bio-Rad). Quantitative real-time PCR (qPCR) reactions were conducted in triplicates with a total volume of 20 μl per reaction using: 2 μl cDNA, 10 μl SYBR green PCR master mix (Bio-Rad), and 100 pmol of forward and reverse primers for each gene of interest (Supplemental Table 3). The following thermal cycler conditions were used: 10 m at 95 °C followed by 35 cycles of 15 s at 95 °C, 30 s at 55–58 °C, and 30 s at 72 °C. mRNA levels of target genes were normalized to human β-actin for each sample, and the fold-change in mRNA postinfection was compared to the uninfected control.

### Inhibitors

MCC950, an inhibitor of NLRP3, was obtained from Calbiochem (256373-96-3) and was added to a final concentration of 10 μM during the course of 3 h in vitro infections. GSK872, a RIP3 kinase inhibitor, was obtained from Glixx Laboratories (GLXC-03990) and was used at a concentration of 5 μM during the course of the 3 h infections. Z-IETD-FMK, a caspase-8 inhibitor, was obtained from Apexbio (B3232) and was used at a concentration of 10 μM during the course of the 3 h infections. THP-1 macrophages were pre-treated with GSK872 and z-IETD-FMK for 2 h prior as well as treated over the course of infections. Ralimetinib, an inhibitor of p38 MAPK, was obtained from Selleckchem (S1494) and used at a final concentration of 0.1 μM during 3 h in vitro infections. CFTR(inh)-172, a selective CFTR inhibitor, was obtained from Sigma (C2992), and was used at a concentration of 10 μM overnight prior to in vitro infections as well as during the 3 or 6 h infection.

### Statistical analyses

GraphPad Prism 8 (GraphPad Software, CA, USA) was used for statistical analyses. Means were compared using either two-sided unpaired Student’s *t*-test or Mann–Whitney *U* test for comparing two groups, with the Shapiro–Wilk test used for verifying normality where applicable and the *F*-test used to test for equality of two variances. For normally distributed data that had unequal variances, Welch’s *t*-test was performed. One-way analysis of variance (ANOVA) (with Dunnett’s multiple comparison tests) was used for comparing multiple groups, with the Brown–Forsythe test used to test for equality of variances. Simple linear regressions were conducted to determine correlations between percent cell viability and the production of cytokines. Densitometric analyses were completed using ImageJ 1.x. The level of significance was set at *P* < 0.05. The following *P* value codes were used for all figures: *P < 0.05, **P < 0.01, ***P < 0.001, ****P < 0.0001.

## Results

### *P. aeruginosa* isolates from chronically infected CF patients induce relatively little macrophage and bronchial epithelial cell death but higher cytokine expression compared with early isolates

**Fig. 1:**
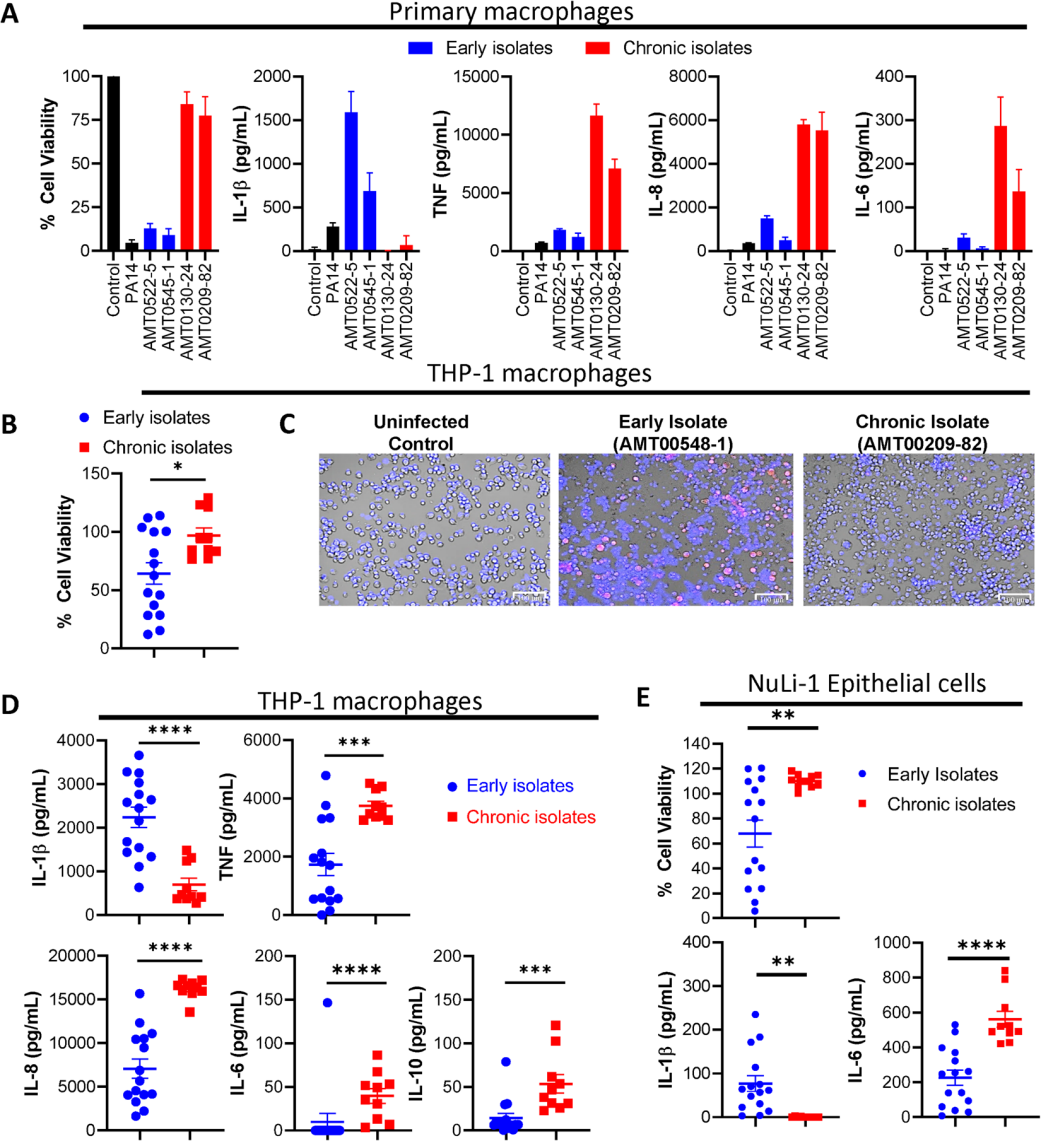
*P. aeruginosa* isolates from chronic CF infections induce less cell death but increased expression of TNF, IL-8, and IL-6 compared with early isolates. Early isolates were defined as isolates from the first positive *P. aeruginosa*-positive sputum culture, while chronic isolates were from patients who had 4 years of positive sputum cultures. Primary human macrophages were seeded at 50,000 cells/well and infected with the indicated early and chronic isolates of *P. aeruginosa* from early and chronic infections or PA14 (10 MOI). Cell viability was measured via neutral red assay and cytokine production was measured via ELISA at 3 h postinfection (**A**). THP-1 macrophages were seeded in fresh media at 100,000 cells/well in a 96-well plate and infected with *P. aeruginosa* isolates from CF patients with early or chronic respiratory infections at 1 MOI as described in Methods and in experimental triplicates. At 3 h postinfection, cell viability was evaluated by neutral red assay, and cytokine production was measured in the supernatants by ELISA (**B**, **D**). Student’s t-tests were used to compare the means for cell viability, TNF, and IL-8 while Mann–Whitney *U* tests were used for IL-1β, IL-6, and IL-10. THP-1 macrophages were seeded at 500,000 cells/well and infected with *P. aeruginosa* (early or late isolate) at 10 MOI. At 2 h postinfection, cells were stained with Hoechst/PI to distinguish live (blue) from dead (pink) cells (**C**). NuLi-1 bronchial epithelial cells were seeded at 30,000 cells/well in a collagen-coated 96-well plate and infected with the same cohort of early and chronic *P. aeruginosa* isolates at 100 MOI and in experimental triplicates. Cell viability via neutral red assay and cytokine production in supernatants via ELISA were measured 6 h postinfection (**E**). Mann–Whitney *U* tests were used to compare IL-1β and IL-6 while Welch’s *t*-test was used to compare the means for cell viability. Mean ± SD of triplicates of a representative, or a pool of >3 experiments are shown. (*P < 0.05, ***P < 0.001, ****P < 0.0001).

We obtained *P. aeruginosa* isolates from CF patients who either had their first positive sputum culture for *P. aeruginosa* (i.e., early isolates) (*n* = 15 patients), or at least a 4-year history of positive sputum cultures (i.e. chronic isolates) (*n* = 10 patients) (Supplemental Table 1). We first generated primary human macrophages from the PBMCs of healthy volunteers and infected them with PA14 or select clinical isolates. PA14 and PAO1 are commonly used reference strains for pathogenesis studies, which were originally isolated from burn/wound patients^15,16^. PA14 and early clinical isolates induced greater levels of cell death and IL-1β expression, but poor expression of TNF, IL-8, and IL-6 (Fig. 1A). We used the THP-1 macrophage cell line to simultaneously test more bacterial samples obtained from CF patients. As with primary human macrophages, infections of THP-1 macrophages in vitro with early or chronic isolates of *P. aeruginosa* elicited significant differences in host cell death: Isolates from chronic infections induced less cell death and lower IL-1β expression than did early isolates (Fig. 1B–D). In contrast, the expression of other inflammatory cytokines TNF, IL-6, and IL-8 was increased upon infection by chronic infection isolates compared to early isolates (Fig. 1D). Expression of the anti-inflammatory cytokine IL-10 was also increased during infections with chronic isolates (Fig. 1D). Infection of NuLi-1 bronchial epithelial cells with early isolates also induced more cell death than infections with chronic isolates (Fig. 1E). As with infection with THP-1 macrophages, NuLi-1 cells infected with chronic *P. aeruginosa* isolates induced less IL-1β expression but more IL-6 expression (Fig. 1E), while the expression of TNF and IL-10 were undetectable (data not shown). Given how the NuLi-1 bronchial epithelial cells failed to express detectable levels of inflammatory cytokines, THP-1 macrophages were predominantly used for further study.

Isolates from early infections induced cell death as early as 2 h postinfection (Fig. S1A). For isolates that induced high levels of THP-1 macrophage cell death, the expression of TNF and IL-8 was limited compared to those isolates that did not induce a high magnitude of cell death. THP-1 macrophages were also exposed to heat-killed bacteria, and we observed that the differences in cell death and cytokine production were reduced (Fig. S1B).

We also used a collection of *P. aeruginosa* isolates from a second cohort of CF patients, where the isolates were obtained from patients longitudinally (Supplemental Table 2). We observed that later isolates reflecting chronic infection stages from longitudinally collected and clonally related *P. aeruginosa* isolates induced less cell death and IL-1β but increased expression of TNF and IL-8 (Fig. S1C).

### Chronic *P. aeruginosa* isolates from CF patients activate inflammasomes poorly

**Fig. 2:**
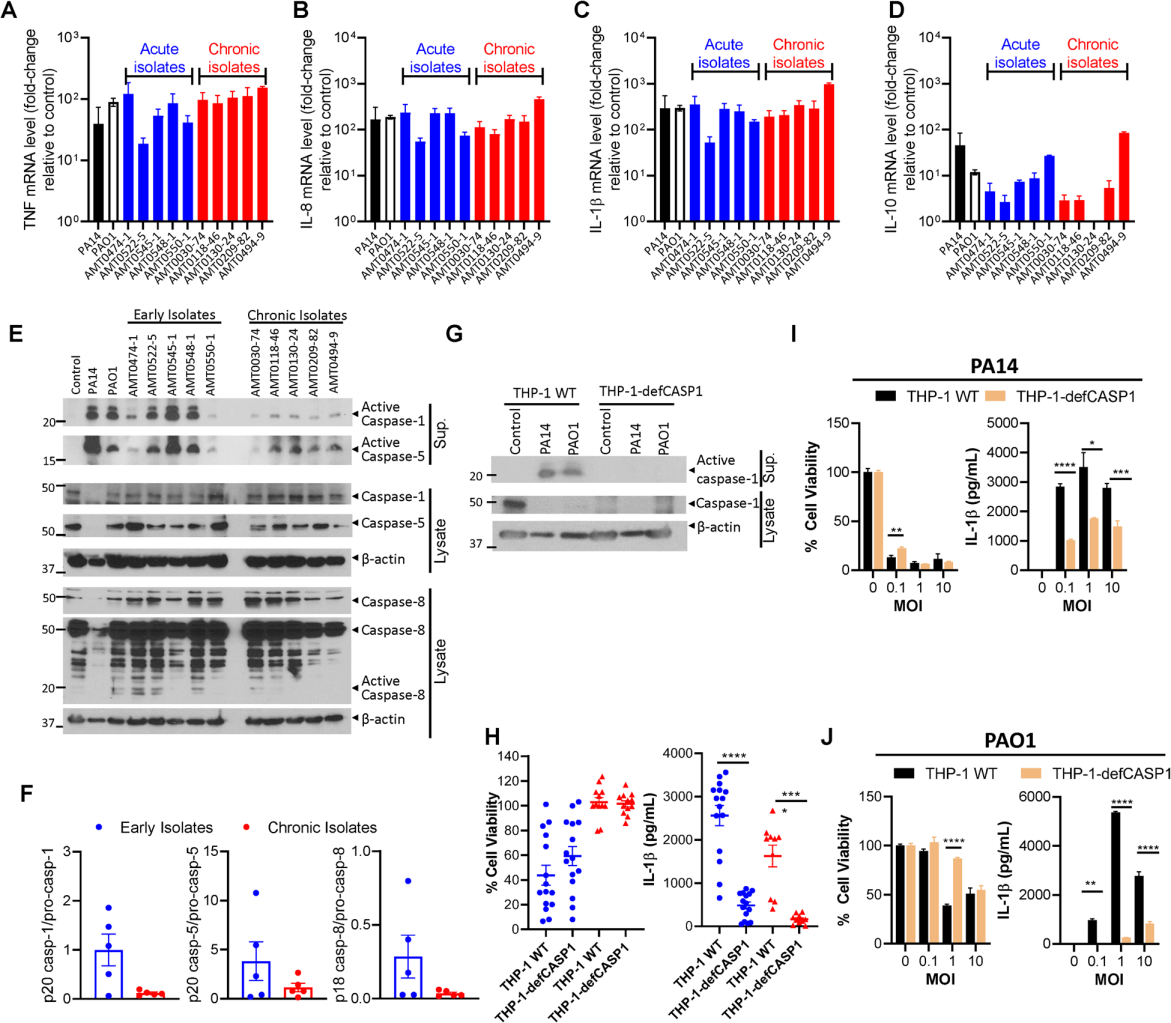
Cell death induced by *P. aeruginosa*, but not IL-1β expression, is independent of caspase-1. THP-1 macrophages were seeded at 500,000 cells/well in a 24-well plate. At 1 h postinfection (1 MOI) with the indicated acute and chronic isolates of *P. aeruginosa* from CF patients. RNA was extracted and cDNA was synthesized. qPCR was conducted on samples targeting genes for various cytokines, and mRNA levels were normalized to β-actin (**A**–**D**). THP-1 macrophages were seeded at 500,000 cells/well in a 24-well plate. At 3 h postinfection (1 MOI) with the indicated acute and chronic isolates of *P. aeruginosa* from CF patients, western blotting was performed on cell supernatants or extracts using specific antibodies shown in figure (**E**). Densitometric analysis of western blots shown in panel E was conducted (**F**). THP-1 WT and THP1-defCASP1 were infected with PA14 or PAO1 (1 MOI) and western blotting was performed on supernatants or cell extracts at 3 h postinfection (**G**). THP-1 WT and THP1-defCASP1 were infected with early and chronic *P. aeruginosa* CF isolates (1 MOI) in experimental triplicates. Cell viability was evaluated at 3 h postinfection by neutral red assay, and IL-1β production was measured in supernatants by ELISA (**H**). Mann–Whitney U tests were conducted. THP-1 WT and THP-1-defCASP1 macrophages were infected with PA14 or PAO1 *P. aeruginosa*, and cell viability and cytokine production were measured as mentioned above (**I**, **J**). Mean values were compared by Student’s *t*-tests, except for measured IL-1β for PA14 at 1 MOI and PAO1 at 0.1 MOI, where Welch’s *t*-tests were used. Mean ± SD of triplicates of a representative, or a pool of >3 experiments are shown. (*P < 0.05, **P < 0.01, ***P < 0.001, ****P < 0.0001).

We sought to determine if *P. aeruginosa* isolates from chronic infections induced greater transcription of cytokines or if isolates from early infections interfered with cytokine processing and secretion through inducing rapid host cell death. Using randomly selected clinical isolates to infect THP-1 macrophages (five from early infections and five from chronic infections), we then measured mRNA levels of IL-1β, TNF, IL-8, and IL-10 through quantitative reverse transcription polymerase chain reaction (qRT-PCR) analysis. It was found that at 1 h postinfection, there was no difference between the levels of mRNA induced during infections using early or chronic isolates (Fig. 2A–D), suggesting that any differences in measured cytokines in the supernatant of infected cells may be due to interruptions in cytokine processing.

We previously reported that *P. aeruginosa* induces both inflammasome-dependent and -independent cell death in murine macrophages^14^. We therefore evaluated the involvement of caspase-1 activation in cell death and IL-1β production in human THP-1 macrophages infected with early and chronic CF infection isolates of *P. aeruginosa*. Caspase-1 was potently activated by the two laboratory reference strains PA14 and PAO1 and the early CF infection isolates of *P. aeruginosa* (Fig. 2E, F). In contrast, there was poor activation of caspase-1, caspase-5, and caspase-8 in macrophages infected by the chronic *P. aeruginosa* isolates compared with early isolates (Fig. 2E, F and Fig. S2A). Since caspase-8 promotes inflammasome-dependent and inflammasome-independent cell death, poor activation of caspase-8 by chronic isolates would be predicted to impact cell death of infected cells through multiple mechanisms. We next determined the role of caspase-1 in cell death and IL-1β secretion during infection with early and chronic isolates of *P. aeruginosa* using a THP-1 cell line that is sevenfold deficient in caspase-1 (THP-1-defCASP1). Activation of caspase-1 was detectable in WT THP-1 cells but not in THP-1-defCASP1 cells following infection with PA14 and PAO1 (Fig. 2G). While the early *P. aeruginosa* isolates induced more cell death of THP-1 cells in comparison to chronic isolates, cell death appeared to be primarily mediated by a non-caspase-1 mechanism regardless of whether the cells were infected with early or chronic isolates (Fig. 2H). The activation of caspases, particularly caspase-1, results in the maturation of IL-1β through cleavage of pro-IL-1β. THP-1-defCASP1 cells demonstrated significant reductions in IL-1β expression in comparison to wild-type THP-1 cells upon infection with both early and chronic infection isolates (Fig. 2H). Therefore, while caspase-1 likely does not play a major role in cell death during infection with clinical *P. aeruginosa* isolates from CF patients, canonical inflammasome activation appears to play a role in IL-1β maturation and secretion. Furthermore, we observed that reducing the availability of active caspase-1 reduced the observed cell death of THP-1 cells at lower MOIs and more appreciably for the PAO1 strain (Fig. 2I, J). In contrast to cell death, caspase-1 played a significant role in IL-1β secretion during *P. aeruginosa* infection (Fig. 2I, J).

We also used the same select 5 *P. aeruginosa* isolates from early and chronic infections each to see how host cell death and cytokine production could change with increased infection time. Increasing the length of infection from 3 to 6 h resulted in increased cell death for infections with some of the early isolates (3 out of 5) but not for infections with chronic isolates (4 out of 5) (Fig. S2B). With the increased infection time, IL-1β and TNF levels did not consistently increase at 6 h postinfection compared to 3 h, whereas IL-6 and IL-10 did (Fig. S2D–F). Notably, trends noticed in cell death and cytokine expression previously observed remained relatively stable even with an increased infection time (Fig. S2B–F).

### Induction of IL-1β secretion by *P. aeruginosa* isolates is dependent on NLRP3

**Fig. 3:**
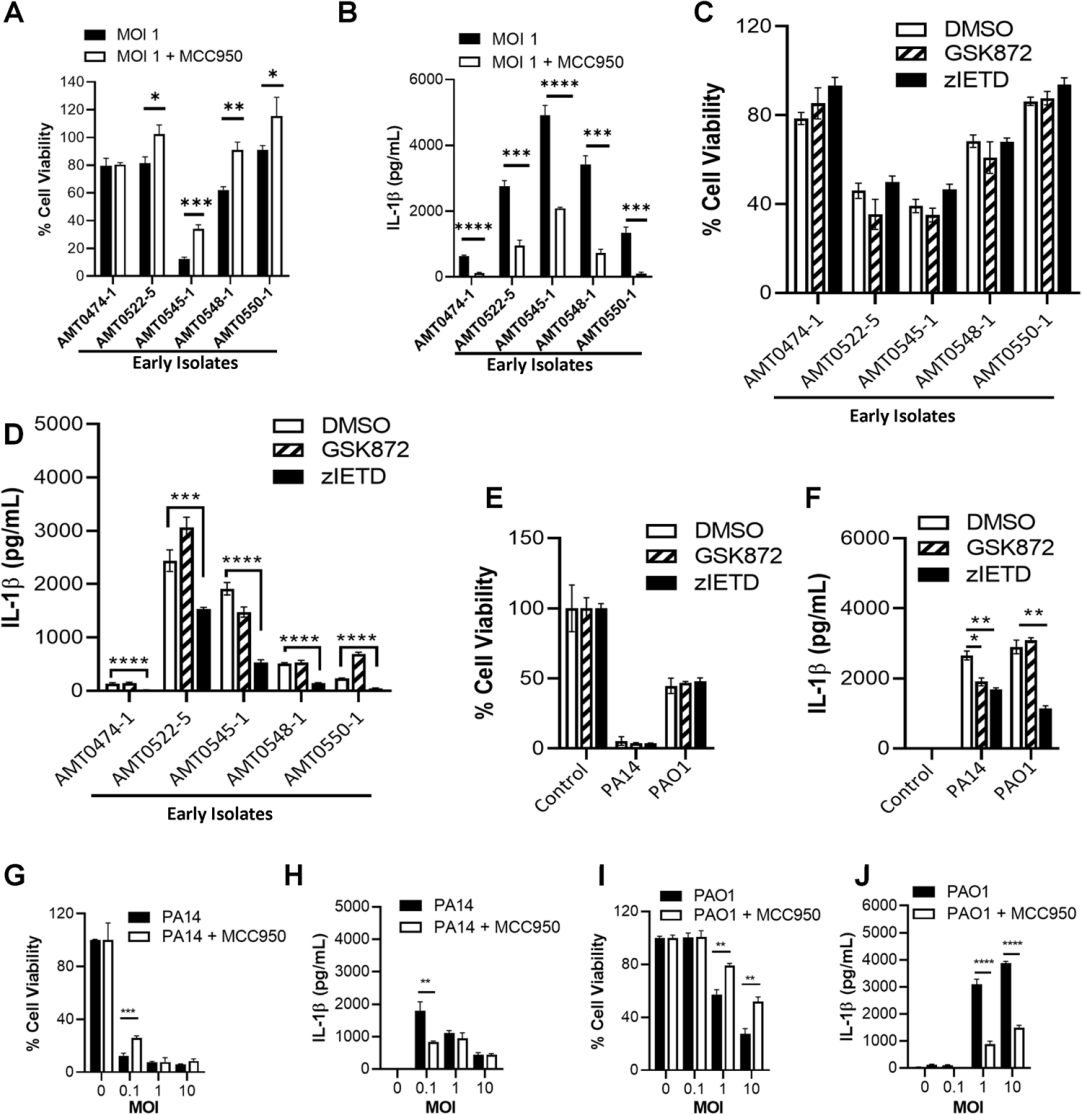
Early CF infection *P. aeruginosa* isolates and PAO1 are partially dependent on NLRP3 to induce cell death and IL-1β production. THP-1 macrophages were seeded as described in Fig. 1. THP-1 macrophages were treated with 10 µM of MCC950 at the time of infection with early *P. aeruginosa* isolates from CF patients and the indicated laboratory reference strains to inhibit NLRP3 activation when infecting THP-1 macrophages. At 3 h postinfection, cell viability was evaluated by neutral red assay (**A**, **G**, **I**) and IL-1β production was measured in cell supernatants by ELISA (**B**, **H**, **J**). THP-1 macrophages were seeded as described in Fig. 1 and pre-treated with 5 µM GSK872 (RipK3 inhibitor), 10 µM z-IETD-FMK (caspase-8 inhibitor) or a DMSO control 2 h prior to infection. Respective treatments were continued during THP-1 macrophage infections with indicated early *P. aeruginosa* isolates or reference strains (1 MOI). At 3 h postinfection, cell viability was evaluated by neutral red assay (**C**, **E**) and IL-1β production was measured in cell supernatants by ELISA (**D**, **F**). Mean ± SD of experimental triplicates are shown. Student’s *t*-tests (**A**, **B**, **G**–**J**) and one-way ANOVAs followed by Dunnett’s multiple comparisons tests (**D**, **F**) were conducted. Mean ± SD of triplicates of a representative experiment are shown. (*P < 0.05, **P < 0.01, ***P < 0.001, ****P < 0.0001).

We used the NLRP3 inhibitor MCC950 to evaluate the role of the NLRP3 inflammasome in the induction of cell death and IL-1β secretion by THP-1 cells. MCC950 inhibits both the canonical and non-canonical NLRP3 inflammasome activation by specifically preventing oligomerization of the ASC scaffold protein^18^. We found that the inhibition of NLRP3 reduced the induction of both cell death (4 out of 5 isolates) and IL-1β expression (5 out of 5 isolates) by early isolates (Fig. 3A, B). These results indicate that NLRP3 promotes cell death and IL-1β expression during macrophage infection by clinical *P. aeruginosa* isolates. To assess the role of necroptosis and apoptosis during *P. aeruginosa* infections, we pre-treated THP-1 macrophages with GSK872 (an inhibitor of RipK3) or z-IETD-FMK (a caspase-8 inhibitor) for 2 h prior to and continued treatment during infections with select clinical isolates from early infections. We found that while neither inhibitor impacted cell death, inhibition of caspase-8 reduced IL-1β expression while GSK872 reduced TNF expression for 3 isolates (Fig. 3C, D and Fig. S3A). This further adds evidence that THP-1 macrophages can engage multiple mechanisms to induce IL-1β expression during *P. aeruginosa* infections.

Similar trends were observed when using laboratory reference strains PA14 and PAO1. Inhibition of caspase-8 did not impact cell death or TNF expression induced by either reference strain but did reduce IL-1β expression (Fig. 3E, F and Fig. S3B). GSK872 also did not impact cell death and appeared to impact TNF and IL-1β expression for infections with PA14 (Fig. 3E, F and Fig. S3B). Furthermore, inhibition of NLRP3 reduced the level of induced cell death more consistently for PAO1 than for PA14 (Fig. 3G, I). We also found that the secretion of IL-1β (Fig. 3H, J), but not TNF (Fig. S3A, B), was consistently reduced by cells treated with the NLRP3 inhibitor.

### Chronic isolates of *P. aeruginosa* strongly activate NF-κB and MAPK than early isolates

**Fig. 4:**
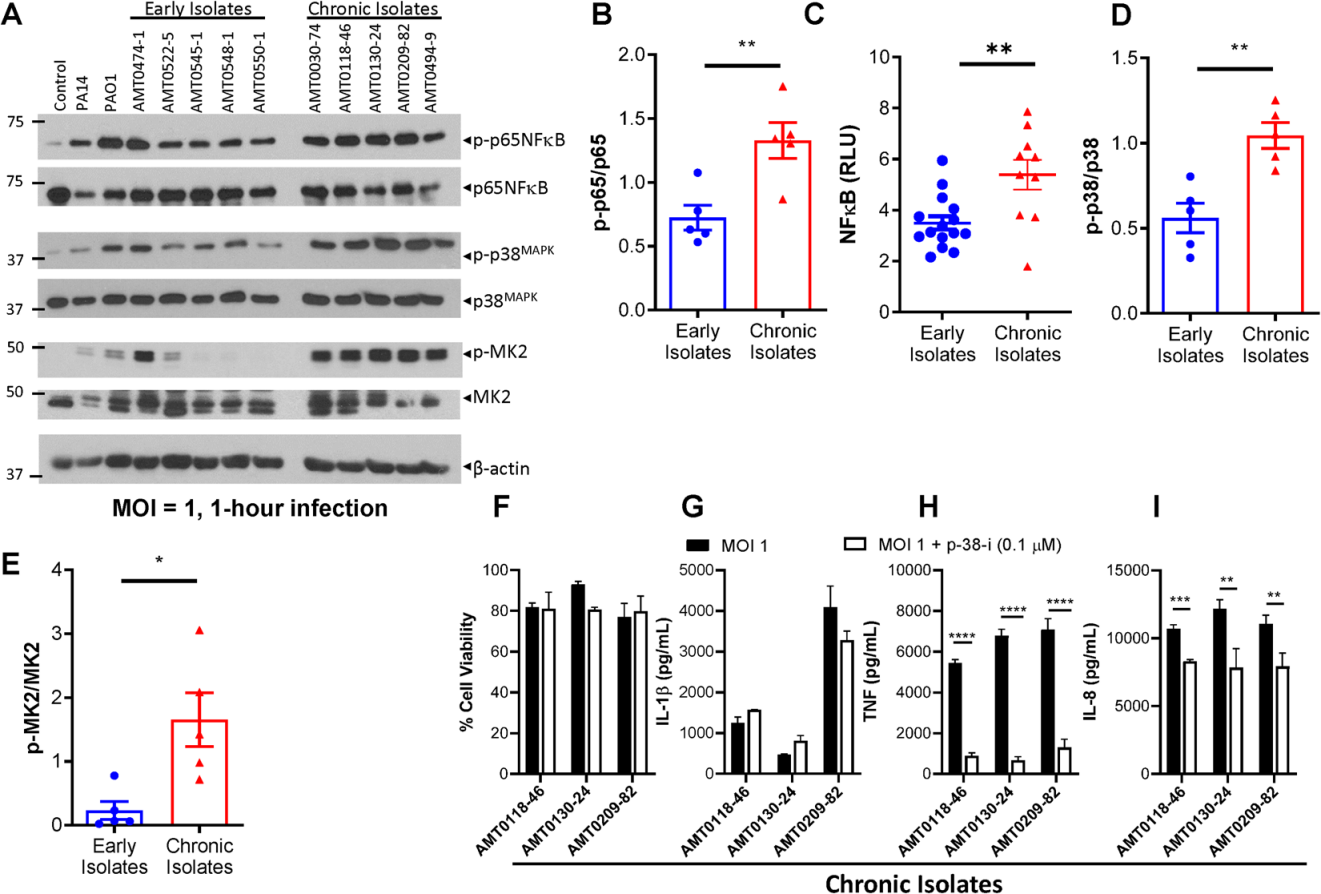
Chronic CF infection *P. aeruginosa* isolates induce increased activation of NF-κB and MAPK more than the early infection isolates. THP-1 macrophages were seeded at 500,000 cells/well in a 24-well plate. Immunoblotting was conducted on lysates collected at 1 h postinfection with the indicated CF *P. aeruginosa* isolates at 1 MOI (**A**). Densitometric analysis of western blots shown in panel A was performed (**B**, **D**, **E**). THP-1 Lucia™ NF-κB cells were differentiated and incubated with 1 MOI of heat-killed early or chronic CF *P. aeruginosa* isolates in experimental triplicates. At 3 h, supernatant luciferase activity (relative light units, RLUs) was measured to assess NF-κB activation (**C**). THP-1 macrophages were seeded at 100,000 cells/well in a 96-well plate. Ralimetinib, a p38 MAPK inhibitor, was added during infections with select chronic *P. aeruginosa* isolates from CF patients to a final concentration of 0.1 µM. Cell viability via neutral red assay (**F**) and cytokine production via ELISA (**G**–**I**) were then measured 3 h postinfection. Mean ± SD of triplicates of a representative experiment are shown. Mean values were compared by Student’s *t*-tests. (*P < 0.05, **P < 0.01, ***P < 0.001, ****P < 0.0001).

Immunoblotting of THP-1 cell extracts at 1 h postinfection revealed that the increase in cytokine expression correlated with enhanced activation of the NF-κB and p38 MAPK pathways by the chronic isolates of *P. aeruginosa* (Fig. 4A, B, D). Activation of the p38 MAPK by chronic isolates of *P. aeruginosa* was further validated by increased activation of MK2, the downstream target of p38 MAPK (Fig. 4A, E). Using the THP-1 NF-κB reporter cell line, we observed that the heat-killed chronic isolates stimulated more NF-κB activation than early isolates (Fig. 4C). This difference suggests that chronic isolates of *P. aeruginosa* from CF patients may differ from early isolates in the expression of heat-stable PAMPs that activate the NF-κB pathway in host cells.

Using ralimetinib, a selective inhibitor of p38α and p38β MAPKs, we observed that there was no impact on THP-1 macrophage death and IL-1β secretion (Fig. 4F, G), but the expression of TNF and IL-8 was decreased upon inhibiting p38 MAPK signaling (Fig. 4H, I). These same trends were observed when exposing THP-1 macrophages to ralimetinib during infections with PAO1 (Fig S4A–D).

### Inverse correlation between cell death and expression of inflammatory cytokine by environmental and non-CF isolates of *P. aeruginosa*

**Fig. 5:**
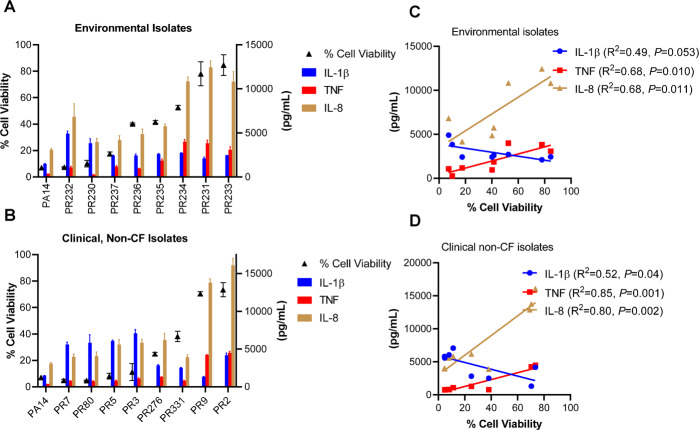
Environmental and clinical, non-CF *P. aeruginosa* isolates also generally exhibit an inverse relationship between induction of cell death and TNF and IL-8 expression. THP-1 macrophages were seeded as described in Fig. 1. Environmental *P. aeruginosa* isolates from river water (**A**) and clinical *P. aeruginosa* isolates from non-CF patients (i.e., blood, wound, and urinary tract infections) (**B**) were used to infect THP-1 macrophages at 1 MOI. Cell viability and IL-1β, TNF, and IL-8 production were measured at 3 h postinfection as described in Fig. 1. Simple linear regression analyses were conducted to determine the relationship between cell viability and cytokine expression for infections with (**C**) environmental and (**D**) non-CF isolates. Mean ± SD of triplicates of a pool of >3 experiments are shown.

We sought to determine how THP-1 macrophages would respond to infection by *P. aeruginosa* collected from the environment (i.e., river water) and non-CF clinical infections (e.g., wounds, urinary tract infections) (Supplemental Table 4). We observed that isolates that induced greater cell death failed to elicit enhanced expression of TNF and IL-8 (Fig. 5A–D). Therefore, the inverse relationship between the magnitude of host cell death and select proinflammatory cytokine expression applies generally to *P. aeruginosa* macrophage infections.

### Mutants of *P. aeruginosa* provide mechanistic insights into the inverse relationship between cytokine expression and macrophage cell death

**Fig. 6:**
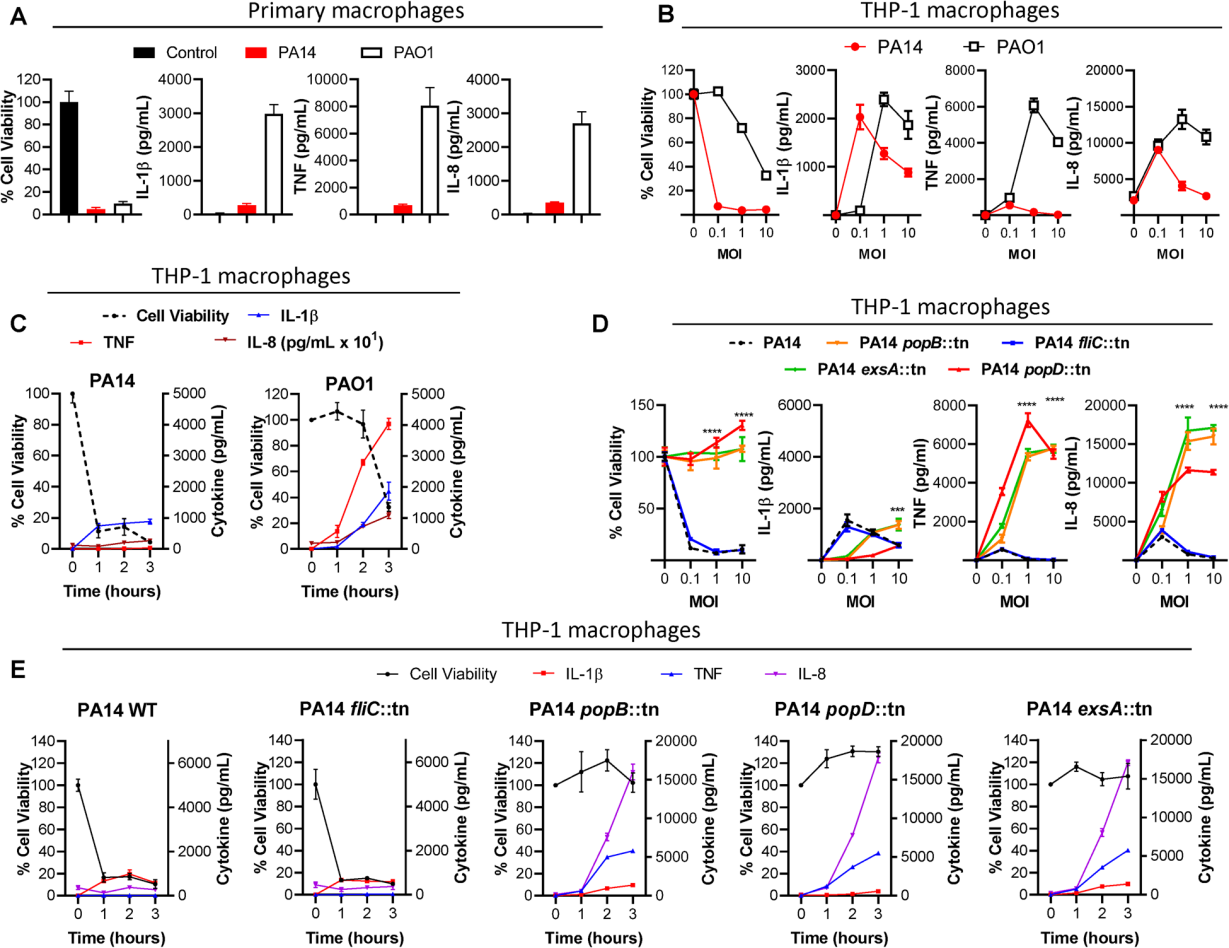
PA14 mutants with impaired T3SS induce less cell death but higher expression of TNF and IL-8 compared to wild-type PA14. Primary human macrophages were seeded at 50,000 cells/well and infected with PA14 or PAO1 at MOI 10. Cell viability via neutral red assay and cytokine production via ELISA were measured 3 h postinfection (**A**). THP-1 macrophages were seeded as described in Fig. 1. In vitro infections were conducted with wild-type PA14 and PAO1, and the various transposon mutants (*fliC*::tn, *popB*::tn, *popD*::tn, and *exsA*::tn) of PA14. Cell viability was measured by neutral red assay and cytokine production was evaluated by ELISA at 3 h (**B**, **D**) or at 1-h intervals (MOI 10) (**C**, **E**). Mean ± SD of triplicates of a representative, or a pool of >3 experiments are shown. One-way ANOVAs were conducted to compare mean values at MOIs of 1 and 10. (****P < 0.0001).

We infected macrophages with *P. aeruginosa* reference strains and mutants of varying virulence in the PA14 background. PA14 is a highly virulent strain of *P. aeruginosa*, while PAO1 is moderately virulent^19^. Some genetic differences between PA14 and PAO1 are predicted to impact mechanisms of host cell death, including the presence in PA14 but not PAO1 of the *exoU* gene^19^, which mediates caspase-1-independent cell death, and the deletion of the *ladS* gene, resulting in enhanced type three secretion system (T3SS) activity. In comparison, cell death by PAO1 is likely to be predominantly inflammasome-dependent. As expected, PA14 induced potent cell death and poor expression of inflammatory cytokines in contrast to PAO1 which induced reduced cell death but an enhanced expression of inflammatory cytokines when infecting primary human macrophages (Fig. 6A) and THP-1 macrophages (Fig. 6B). THP-1 macrophage cell death when infected with PA14 occurred relatively early, nearing 90% cell death after 1 h, with only limited TNF and IL-8 expression (Fig. 6C). In contrast, cell death induction by PAO1 was both delayed and reduced compared with PA14, and the expression of TNF and IL-8 was greater (Fig. 6C). Similar results were also observed with the NuLi-1 bronchial epithelial cells infected with either PA14 or PAO1 (Fig. S5A).

Infection with the flagellar mutant PA14 *fliC*::tn induced similar levels of THP-1 macrophage cell death as wild-type PA14 (Fig. 6D). PA14 T3SS mutants *popB*::tn and *popD*::tn (translocator proteins of T3SS) and *exsA*::tn (a T3SS transcriptional regulator) induced minimal cell death (Fig. 6D). Expression of IL-1β correlated positively with cell death at a lower MOI (Fig. 6D). Both wild-type PA14 and PA14 *fliC*::tn potently induced cell death yet poor expression of TNF and IL-8, consistent with the inverse patterns of early cell death and cytokine production (Fig. 6D). PA14 and PA14 *fliC*::tn in comparison to other transposon mutants also demonstrated extensive THP-1 cell death within 1 h of infection, which resulted in compromised production of TNF and IL-8 by both PA14 and PA14 *fliC*::tn (Fig. 6E). Cell viability and cytokine expression trends observed among the PA14 transposon mutants and wild-type PA14 were consistent upon extending the course of in vitro infections from 3 to 6 h (Fig. S5B).

### Inhibition of CFTR does not impact the inverse relationship between cell death and cytokine expression during *P. aeruginosa* infections

**Fig. 7:**
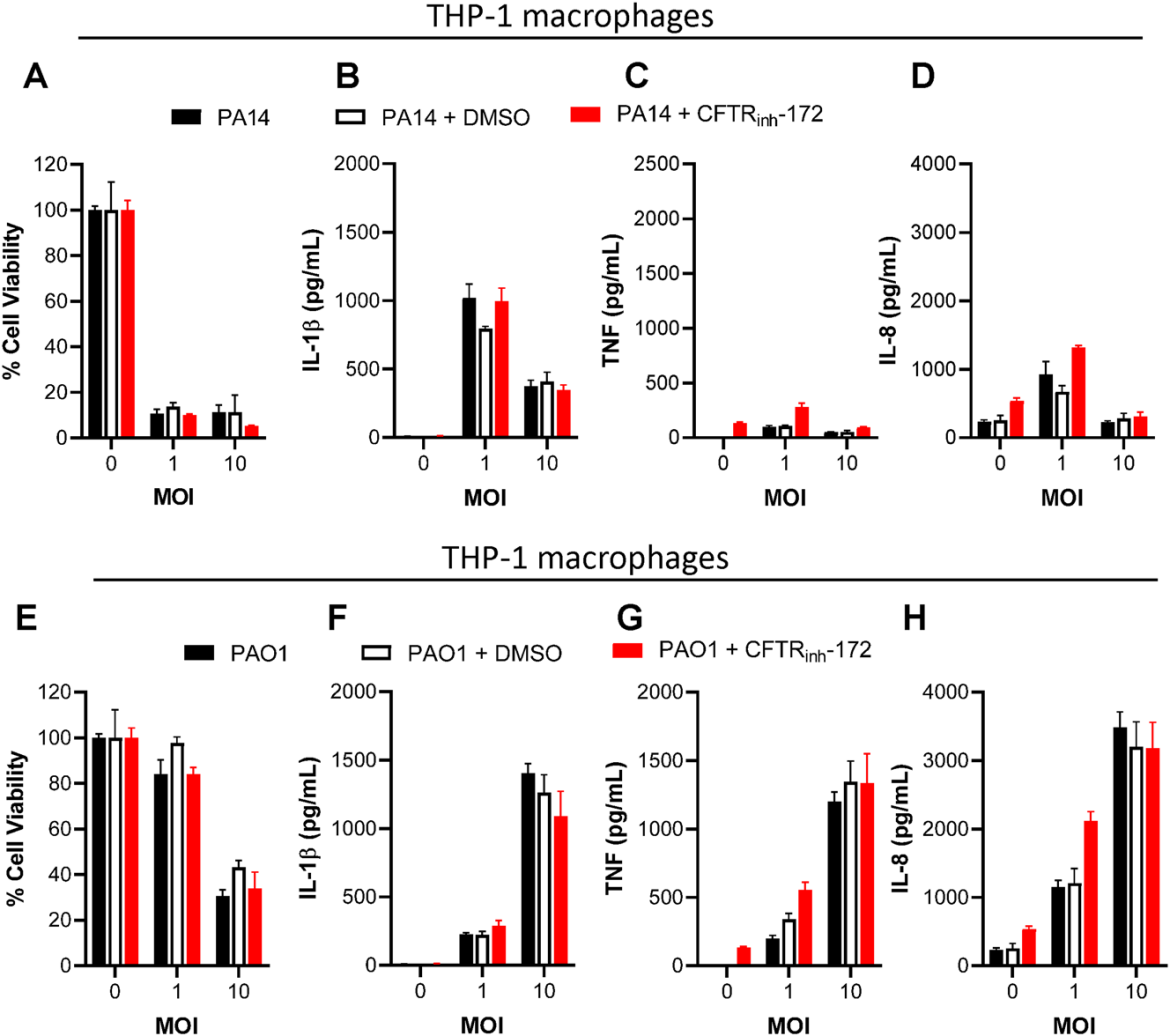
CFTR inhibition in THP-1 macrophages does not impact cell death or cytokine production. THP-1 macrophages were seeded as described in Fig. 1 with the addition of either a DMSO vehicle control or 10 µM CFTR(inh)-172 overnight. In vitro infections were conducted with PA14 (**A**–**D**) or PAO1 (**E**–**H**) with the addition of either treatment. At 3 h postinfection, cell viability was evaluated by neutral red assay (**A**, **E**) and cytokine production was measured by ELISA (**B**–**D**, **F**–**H**). Mean ± SD of triplicates of a representative experiment are shown.

We sought to investigate if inhibition of CFTR function impacted inflammatory cell death and cytokine expression by macrophages and epithelial cells. We treated THP-1 macrophages overnight with the CFTR-selective inhibitor (CFTR(inh)-172) using a previously reported concentration of 10 µM^20–23^ and infected them with PA14 or PAO1. No clear impact of the CFTR inhibitor was observed in cell death or cytokine expression during PA14 (Fig. 7A–D) and PAO1 (Fig. 7E–H) infections. Furthermore, greater expression of IL-1β, TNF, and IL-8 was still observed during infection with PAO1 than PA14 with or without the presence of CFTR(inh)-172. The same treatments were applied to the bronchial epithelial cells. As with THP-1 macrophages, there were no significant differences between treatment groups during infections with PA14 (Fig. S6A) or PAO1 (Fig. S6B) infections.

### Inverse correlation between host cell death and cytokine expression is observed between *P. aeruginosa* and *S. aureus*

**Fig. 8:**
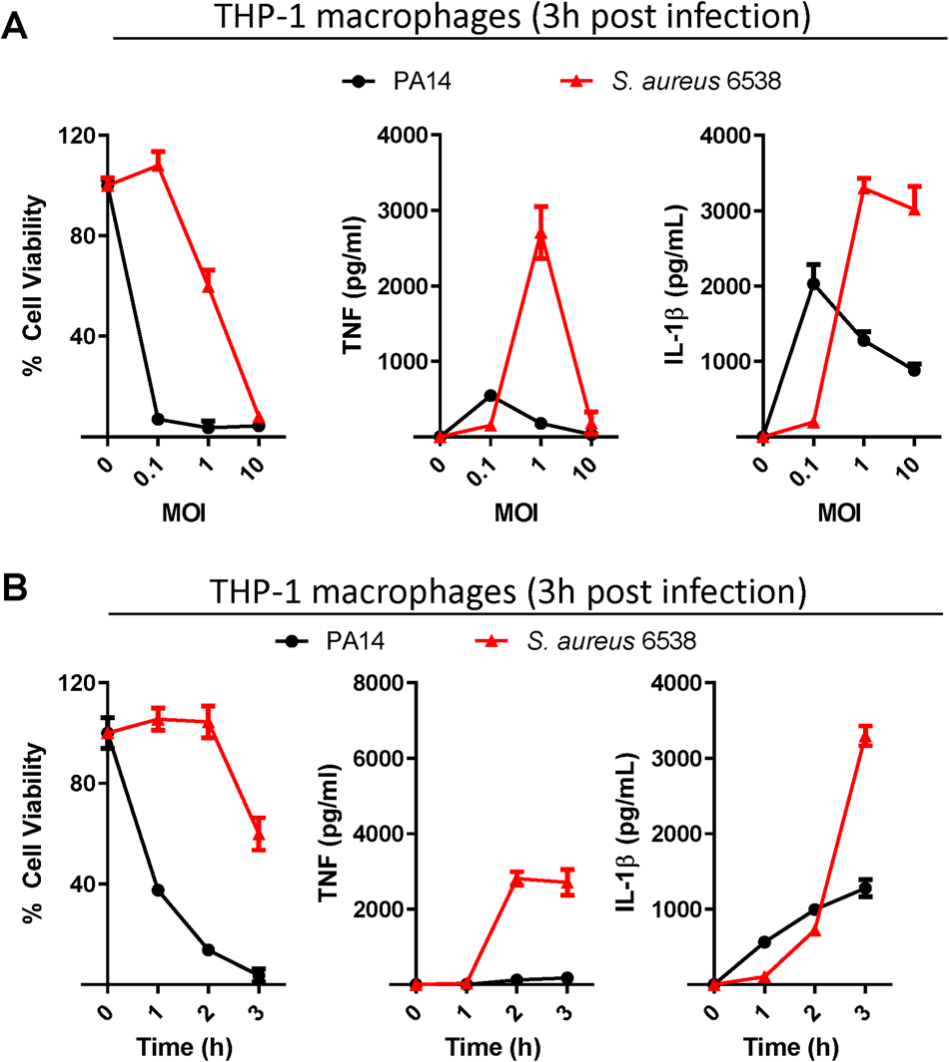
PA14 and *S. aureus* demonstrate an inverse relationship between cell death and cytokine expression. THP-1 macrophages were seeded as described in Fig. 1 and infected with either PA14 or *S. aureus* 6538 at various MOIs. Three hours postinfection, cell viability via neutral red and TNF and IL-1β in the supernatant via ELISAs were measured (**A**). Infections were also performed using an MOI of 1, and cell viability and cytokines were measured at 1-h intervals for up to 3 h (**B**). Mean ± SD of triplicates of a representative experiment are shown.

*Staphylococcus aureus* (*S. aureus*) is another pathogen that often colonizes the lungs of CF patients^24^. Using laboratory reference strains *S. aureus* 6538 and PA14 to infect THP-1 macrophages, we found that *S. aureus* 6538 did not induce cell death as rapidly as PA14, which allowed for higher levels of TNF and IL-1β expression at specific MOIs and over time (Fig. 8). Therefore, it may be that the trends observed in our work may be applicable across different species of bacteria, including those that are frequently seen in CF.

Taken together, these results support a model in which rapid cell death limits the expression of cytokines by the infected cell. Based on our results, it appears that macrophage survival during the initial few hours of infection is critical in determining the extent of cytokine expression.

## Discussion

Opportunistic pathogens infect the lungs of people with CF and adapt to the airway environment^4,9^. *P. aeruginosa* isolates evolve in the lungs of CF patients and display a phenotype of reduced virulence^6,25–28^ to facilitate evasion of host immunity^4^. However, inflammatory cytokines such as TNF and IL-8 are often found to be elevated in the bronchoalveolar lavage of CF patients with ongoing infection^29^. Furthermore, intravenous antibiotic treatment frequently decreases airway inflammation in CF patients^10,29–33^, indicating that despite adaptation in the host, avirulent bacteria that reside in the lungs of CF patients continue to induce significant inflammation. A key question that arises is how these so-called “non-virulent” bacteria promote inflammation and decline in lung function. We have revealed an inverse relationship between cell death and cytokine production in macrophages. While necrotic cell death is generally considered to be inflammatory^34,35^, we propose that the relationship between *P. aeruginosa* adaptation and increasing respiratory pathology is due to persistent cytokine production by phagocytes that survive infection (i.e., by a late-infection, adapted, lower-virulence bacterium). The inverse relationship between the induction of phagocyte death and proinflammatory activity raises questions about the definition of “virulence” as it applies to *P. aeruginosa* in CF and other chronic infections, where both persistence of infection and host inflammation could be considered to contribute to pathogenesis.

Chronic *P. aeruginosa* frequently downregulates the expression of flagellin and T3SS components, which could result in evasion of host immune surveillance^36^. *P. aeruginosa* flagellin binds to the NOD-like receptor (NLR) neuronal apoptosis-inhibitory protein (NAIP), resulting in the activation of the NLRC4 inflammasome, cell death by pyroptosis, and IL-1β secretion^37^. As expression of both T3SS and flagell in are often defective in chronic *P. aeruginosa* isolates^6,14,26,38^, our results indicate that pyroptosis among innate immune cells would be relatively diminished during chronic infections, perhaps facilitating the persistence of *P. aeruginosa* in the CF lung^14^. Using PA14 transposon mutants to infect THP-1 cells, here we found that T3SS components, but not flagellum structural component FliC, were required to induce cell death, consistent with the importance of the T3SS in injecting toxins and virulence factors into host cells leading to cell death of phagocytes.

We found that the activation of caspases-1, -5, and -8 by chronic CF infection isolates of *P. aeruginosa* is relatively poor. In mice, the caspase-5 homolog caspase-11 has been shown to mediate non-canonical inflammasome signaling in response to cytosolic LPS^39^. Whether *P. aeruginosa* LPS is present in the cytosol during macrophage infections, and the role of LPS in activating human caspases-4 and -5, remains to be determined. Caspase-8 promotes cell death by the extrinsic-, the ripoptosome-, and the NLRP3- pathways^40–42^, all of which would be predicted to be diminished during chronic CF infection with *P. aeruginosa*. Thus, the overall program of macrophage cell death would be predicted to be diminished during infections by chronic isolates of *P. aeruginosa* in CF patients compared with early infection isolates.

Cell death is bound to play a key role in influencing pathogen chronicity and inflammation in different infection models. It has been recently reported that the expression of the virulence gene hemolysin-A is modulated in uropathogenic isolates of *E. coli* which impacts the inflammasome activation and bacterial colonization^43^. Bacterial strains that expressed more hemolysin-A induced more cell death and increased bladder colonization^43^. Based on our study, increased cell death would result in poor expression of inflammatory cytokines such as TNF, IL-8, and IL-6 and it is conceivable that poor expression of these cytokines results in a compromised innate immune response leading to increased bacterial colonization.

Our findings are consistent with previous work demonstrating that the mechanism of IL-1β maturation and secretion differs from other cytokine processing pathways. Cytokine secretion by the conventional pathway involves the endoplasmic reticulum (ER)-Golgi route, similar to other secreted proteins that transit the ER and the Golgi complex to undergo post-translational modifications prior to exocytosis^44,45^. The IL-1 cytokine family lacks the secretion signal to be trafficked through this route. Monocytes may engage in any one of a diverse set of pathways to secrete IL-1β, including lysosome exocytosis, microvesicle shedding, and caspase-1-dependent cellular pore formation^44,45^. These mechanisms may be more rapid than the conventional ER-Golgi route^44,45^. Therefore, it may be that in our study, human macrophages are able to engage relatively rapid pathways facilitating IL-1β secretion in spite of early cell death when infected with early infection *P. aeruginosa* isolates. However, under the same circumstances, the expression of other cytokines could be limited by insufficient time for macrophages to engage in more traditional cytokine gene expression, translation, and processing.

TNF is a highly inflammatory cytokine that plays an important role in numerous inflammatory diseases^46^. IL-8 is a proinflammatory and chemoattractant cytokine that helps recruit and activate a variety of immune cells, especially neutrophils^47^. It was interesting to note that IL-1β expression, although reduced, was still detected when using chronic isolates in our study. These findings may be reflective of clinical findings where IL-1β, TNF, and IL-8 are found to be present in BALF^29,48^ and sputum^49^ in patients with CF compared to non-CF controls.

Our study demonstrates that *P. aeruginosa* from chronic infections in CF retains the ability to stimulate the expression of inflammatory cytokines. Previous reports demonstrated an increase in baseline activation of NF-κB and p38 MAPK pathways in CF airway epithelial cells in comparison to wild-type CFTR cells in response to *P. aeruginosa* infections^50–53^. Elevated NF-κB may in turn result in increased transcription of NLRP3 and expression of IL-1β^20,54^. Our findings are reflective of a previous study that demonstrated no increase in IL-1β secretion upon exposing THP-1 macrophages with CFTR(inh)-172^20^, and the use of other cellular models in the literature have yielded inconsistent results^55,56^. However, it is important to note that our described paradigm of host cell death disrupting cytokine processing and secretion appears to apply even when CFTR is inhibited, which has implications for observations of proinflammatory markers seen among CF patients. It has been suggested that there are correlations between elevated cytokine levels in CF patient sputum and clinical parameters, such as declining lung function and lung disease^10,11,57^. Defining how CF pathogens stimulate airway inflammation, and how that activity relates to disease progression, represents a critical step in developing therapies that target these pathways to prevent morbidity in CF and other airway infections^58^. The results of this study reveal a novel mechanism in which an adaptive change of a key chronic infectious pathogen leads to diminished macrophage cell death yet augmented inflammatory cytokine production, indicating that properties that limit virulence to host cells can still result in worse disease.

## Supplementary information

Supplemental figures and tables
