# Supplementary material for: Impairment in inflammasome signaling by the chronic Pseudomonas aeruginosa isolates from cystic fibrosis patients results in an increase in inflammatory response
Source: Cell Death Dis. 2021 Mar 4;12(3):241. doi: 10.1038/s41419-021-03526-w (PMC7933143; doi:10.1038/s41419-021-03526-w)
Supplement: Supplementary file 1 — Supplemental figures and tables [file 41419_2021_3526_MOESM1_ESM.pdf]

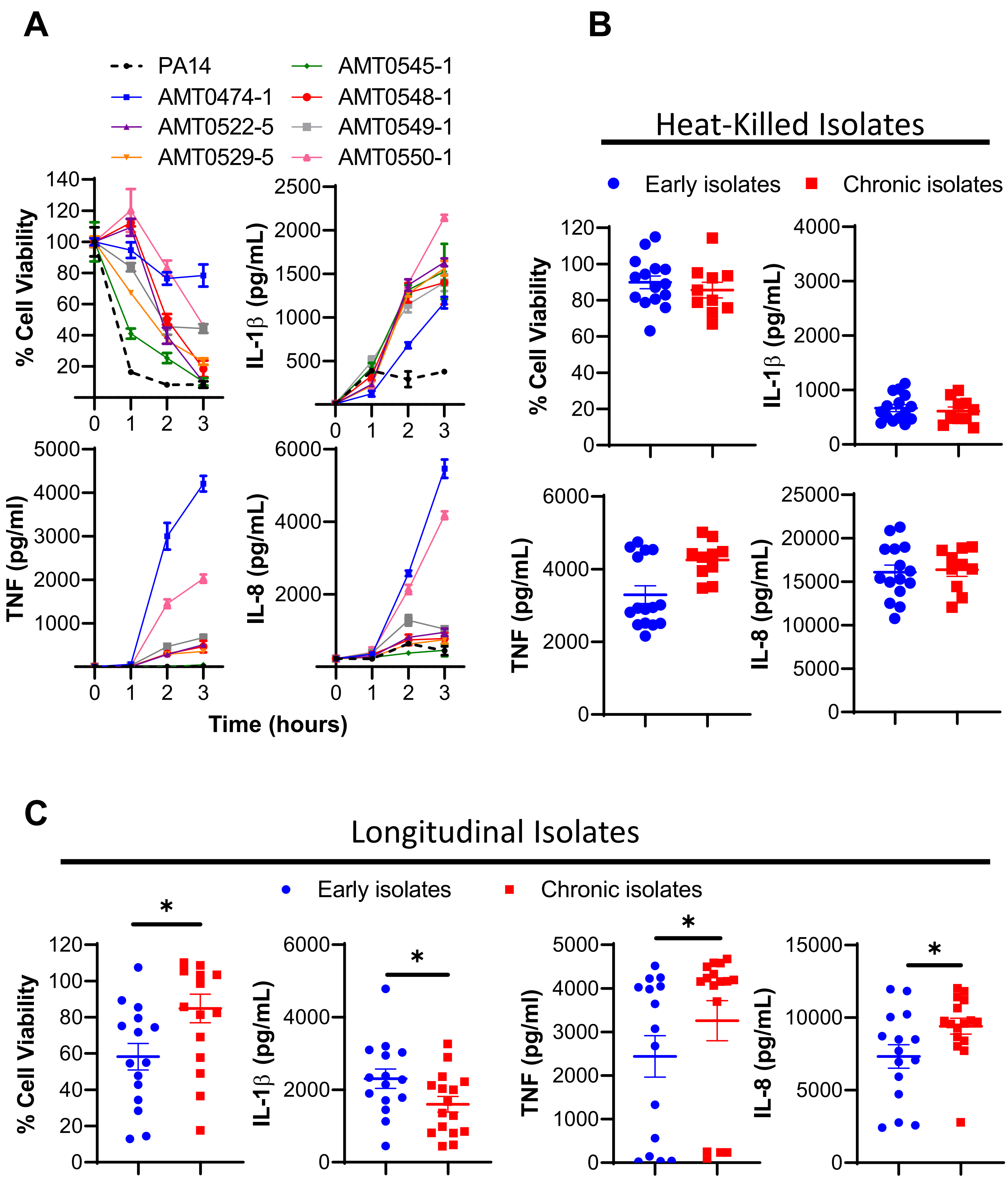

Fig. S1. Early CF infection *P. aeruginosa* isolates induce death as early as 1 hour post infection. THP-1 macrophages were seeded as described in Fig. 1 and infected with the indicated early infection isolates of *P. aeruginosa* (10 MOI). At the time intervals shown, cell viability was evaluated by neutral red assay (**A**). Expression of cytokines was measured in supernatants at various time intervals by ELISA (**A**). The *P. aeruginosa* isolates described in Figure 1 were heat-killed and incubated with THP-1 macrophages as described above. Cell viability was measured via neutral red assay and cytokine production was measured via ELISA at 3 h postinfection (**B**). THP-1 macrophages were seeded as described in Fig. 1 and infected with early and chronic longitudinal isolates of *P. aeruginosa* from CF patients collected at least 1.5 years apart. Infections were performed in experimental triplicates. At 3 h postinfection with live bacteria (1 MOI), cell viability and cytokine assays were conducted as described above (**C**). Student's t-tests were used to compare the means for cell viability and IL-1β, while Mann–Whitney U tests were used to compare TNF and IL-8. Mean ± SD of experimental triplicates are shown. (*$P < 0.05$).

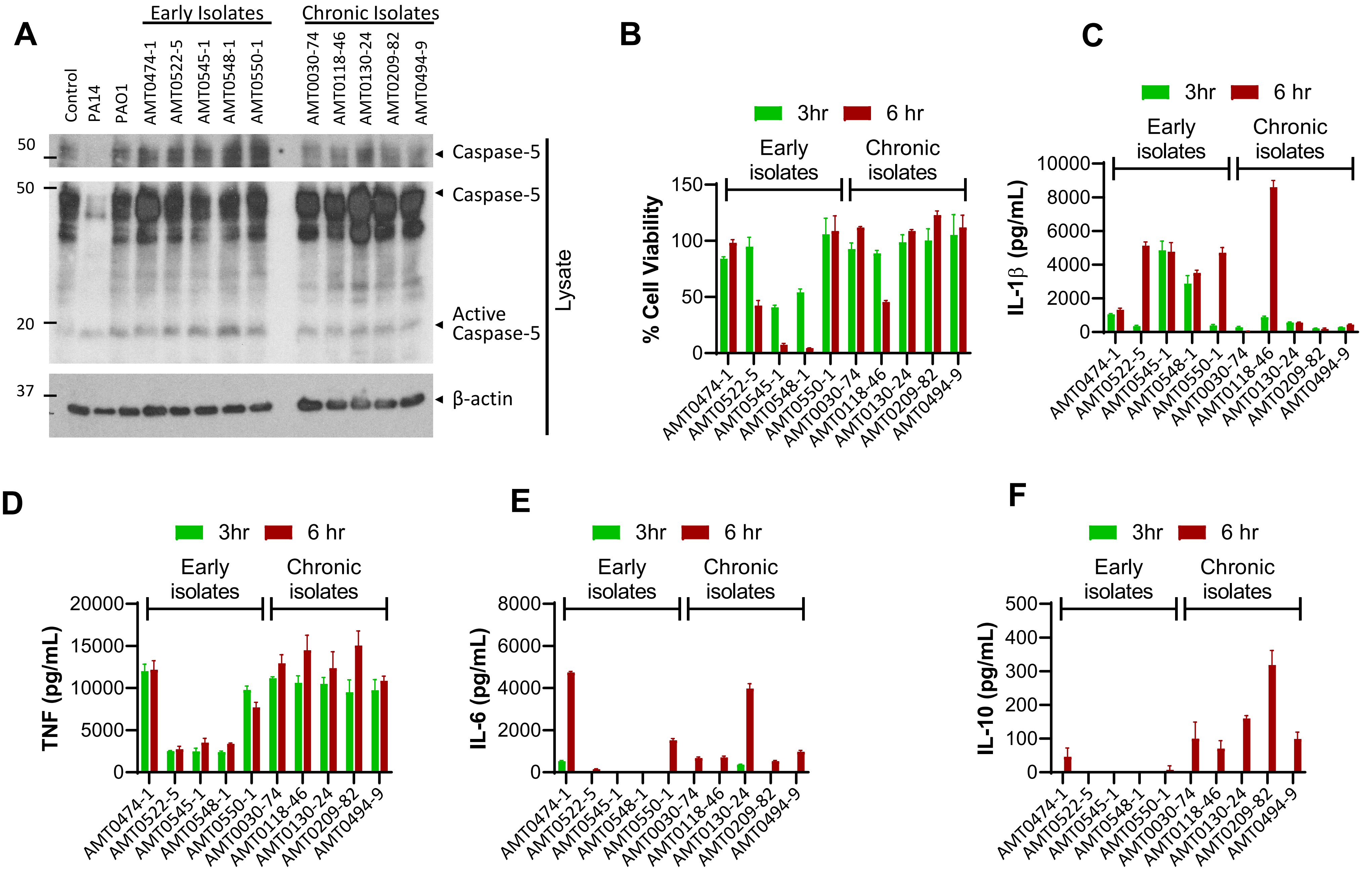

Fig. S2. An inverse relationship between cell death and cytokine expression is still observed with increased time of infection. THP-1 macrophages were seeded at 500,000 cells/well in a 24-well plate and infected with the indicated clinical *P. aeruginosa* isolates (1 MOI). At 3 h postinfection, western blotting was performed on cell extracts using the indicated antibodies (**A**). *In vitro* infections with selected clinical *P. aeruginosa* isolates were conducted (1 MOI). At 3 and 6 h postinfection, cell viability was assessed via neutral red assay (**B**) and cytokine levels were measured in the supernatant using ELISAs (**C-F**). Mean ± SD of experimental triplicates are shown.

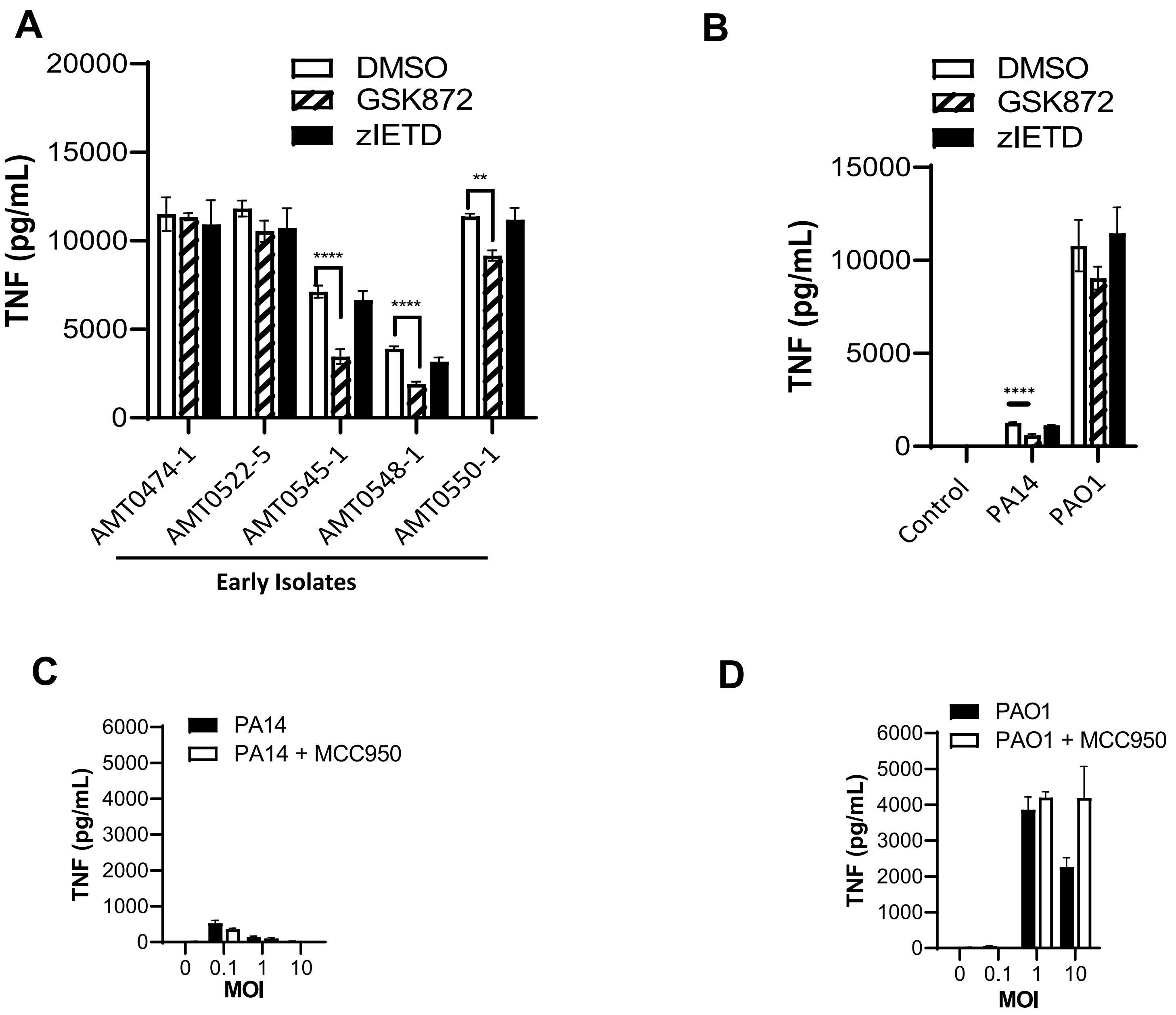

Fig. S3. Inhibition of RipK3 reduces TNF expression during infections with some *P. aeruginosa* strains. THP-1 macrophages were seeded as described in Fig. 1. *In vitro* infections with indicated early *P. aeruginosa* isolates or reference strains were then conducted (1 MOI) with the addition of either 5 µM GSK872, 10 µM z-IETD-FMK, or a DMSO control as described in Fig. 3. At 3 h postinfection, TNF expression was measured via ELISA (**A, B**). One-way ANOVA followed by Dunnett's multiple comparison tests were conducted. THP-1 macrophages were seeded as described in Fig. 1, and *in vitro* infections with reference strains were then conducted at various MOIs with and without the addition of 10 µM MCC950 as described in Fig. 3. At 3 h postinfection, TNF expression was measured via ELISA (**C, D**). Mean ± SD of experimental triplicates from a representative experiment are shown. (**$P < 0.01$, ****$P < 0.0001$).

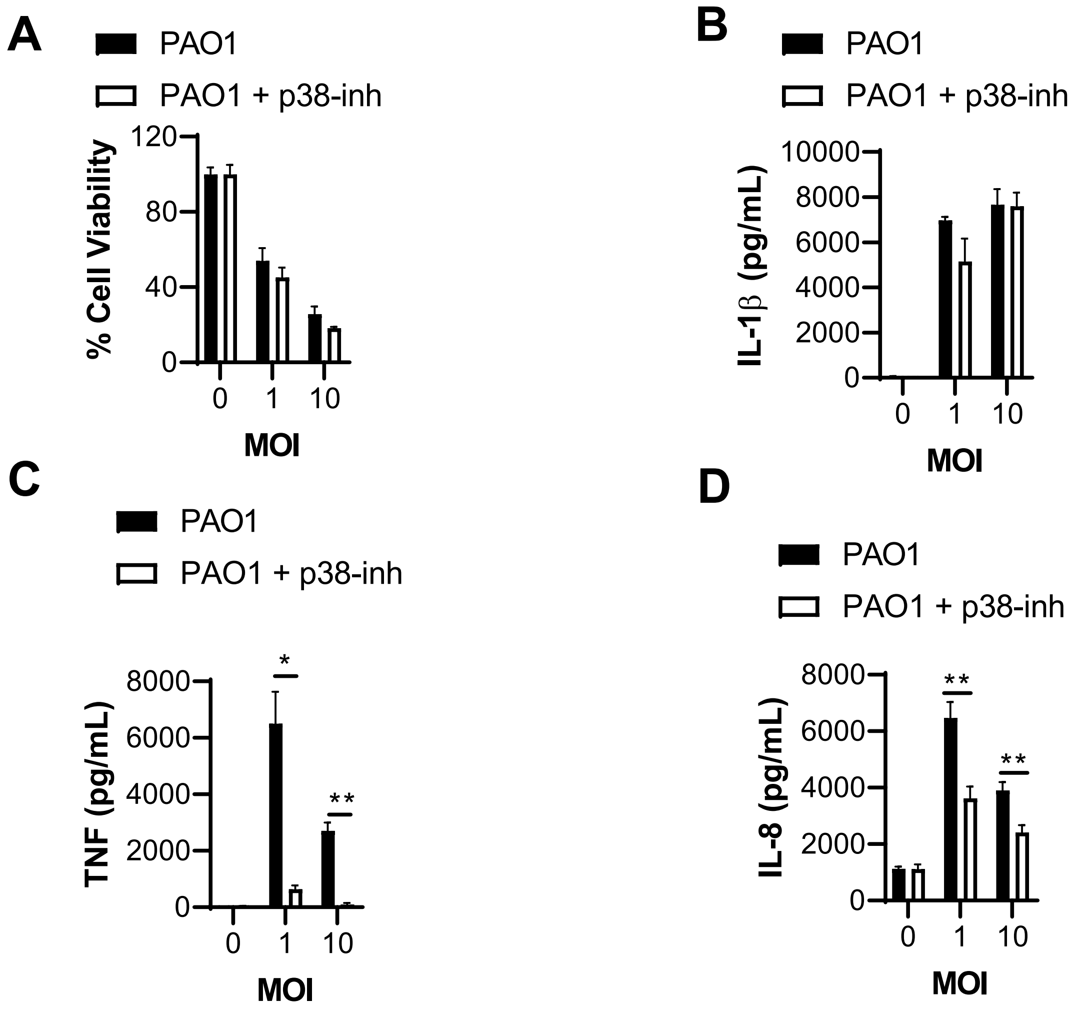

Fig. S4. Inhibition of p38 MAPK significantly reduces TNF and IL-8 expression during infections with PAO1. THP-1 macrophages were seeded as described in Fig. 1. *In vitro* infections with PAO1 were then conducted at MOI 1 and 10 with and without the addition of 0.1 µM ralimetinib, a p38 MAPK inhibitor. At 3 h postinfection, cell viability was evaluated via neutral red assay (**A**) and cytokine expression was measured via ELISA (**B-D**). Mean ± SD of experimental triplicates from a representative experiment are shown. To compare means, Student's t-tests were used except for measured TNF at MOI 1, where Welch's t-test was used. (*$P < 0.05$, **$P < 0.01$).

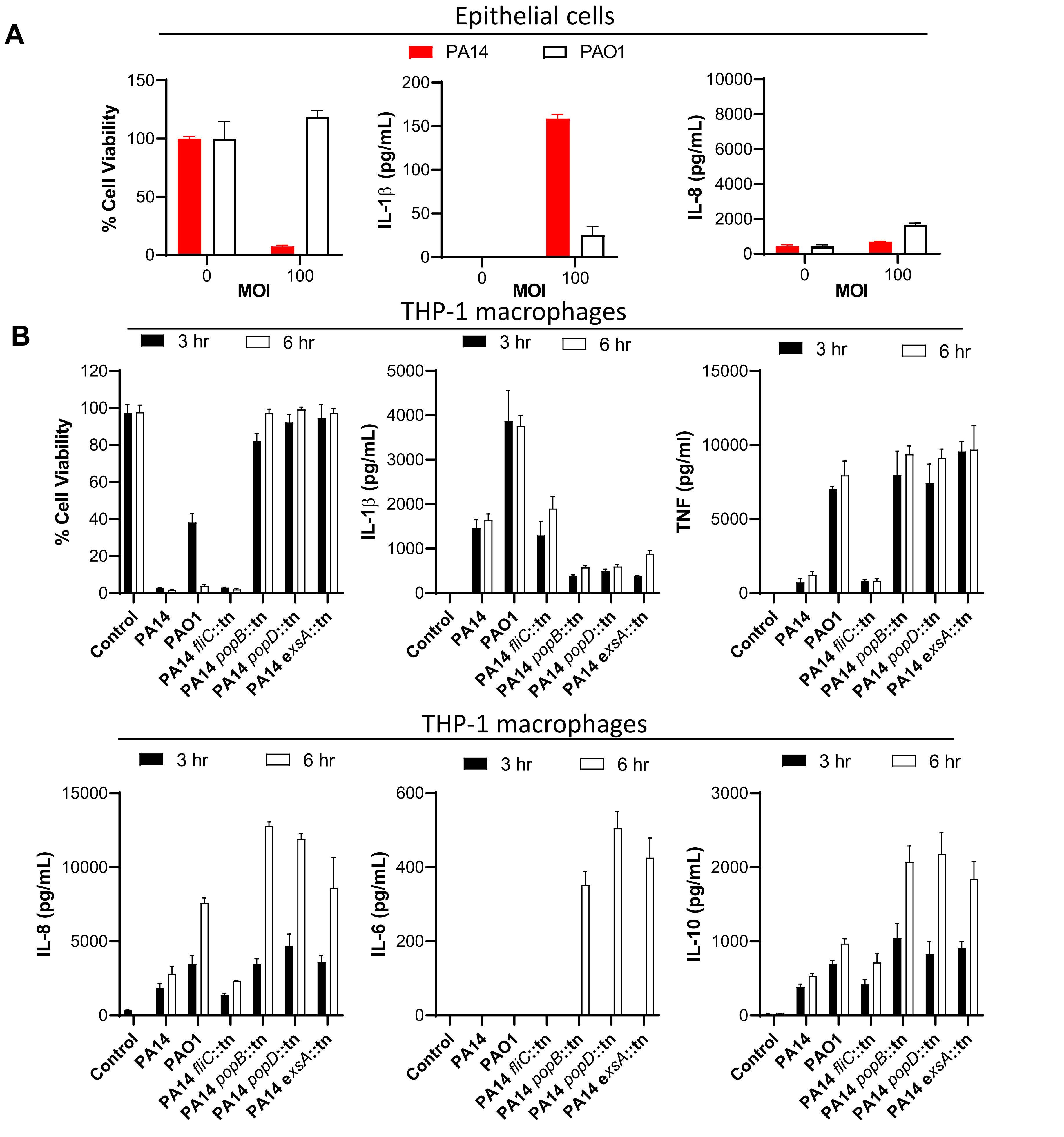

Fig. S5. Inverse patterns of cell death and expression of TNF, IL-8, IL-6, and IL-10 by mutants of *P. aeruginosa*. Bronchial epithelial NuLi-1 cells were seeded as described in Fig. 1 and infected with either PA14 or PAO1 at 100 MOI. Cell viability via neutral red assay and cytokine production via ELISA were measured 6 h postinfection (**A**). THP-1 macrophages were seeded as described in Fig. 1. *In vitro* infections were conducted with reference strains or indicated transposon mutants of PA14 (1 MOI). At 3 h and 6 h postinfection, cell viability was evaluated by neutral red assay. Cytokine production was measured in cell supernatants by ELISA (**B**). Mean ± SD of experimental triplicates from a representative experiment are shown.

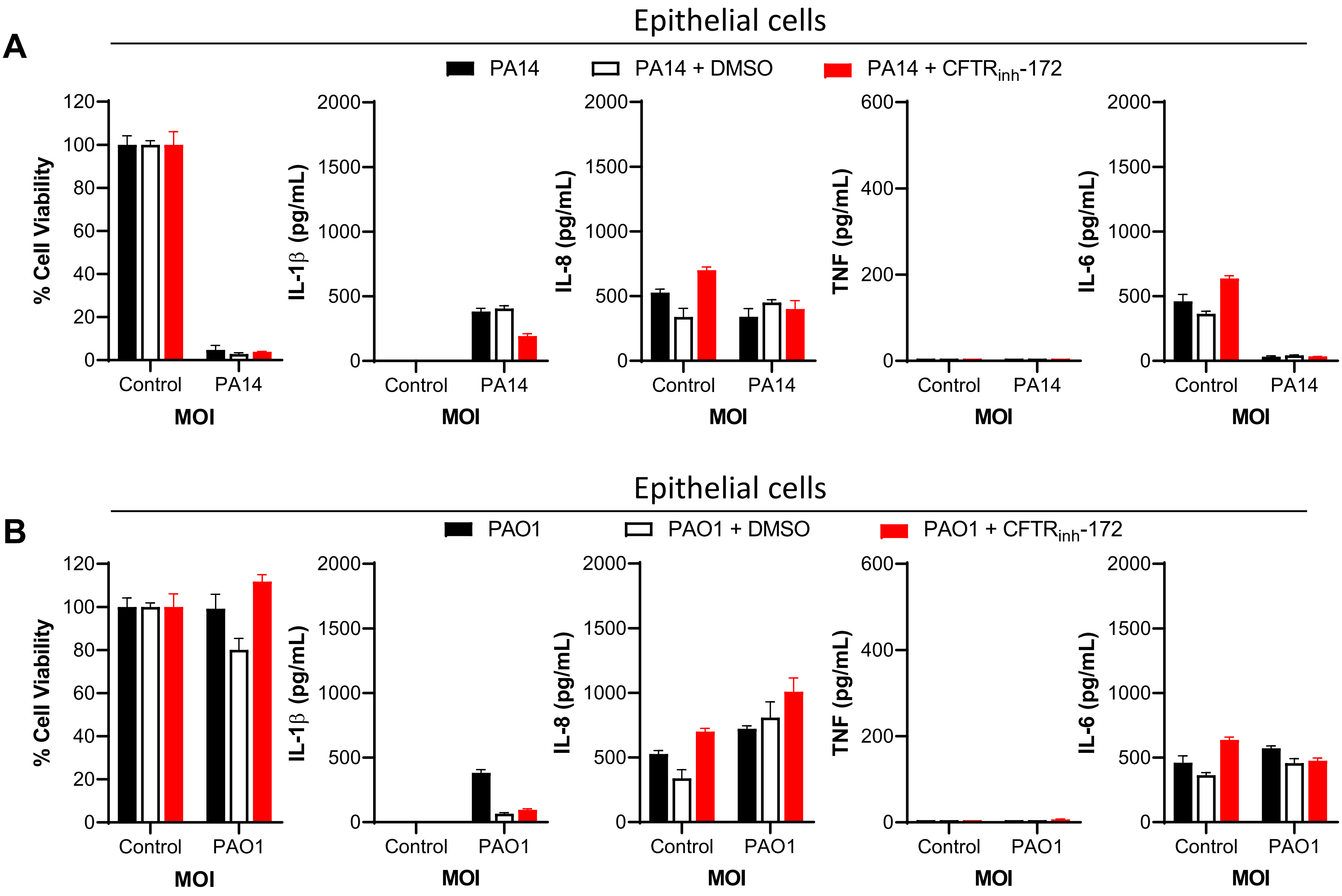

Fig. S6. Inhibition of CFTR in NuLi-1 cells does not impact cell death or cytokine production. NuLi-1 cells were seeded as described in Fig. 1 with the addition of either a DMSO vehicle control or 10 µM CFTR(inh)-172 overnight. *In vitro* infections were conducted with PA14 or PAO1 with the addition of either treatment. At 6 h postinfection, cell viability was evaluated by neutral red assay and cytokine production was measured by ELISA (**A, B**). Mean ± SD of experimental triplicates from a representative experiment are shown.

Supp. Table 1. *P. aeruginosa* isolates from CF patients' with early and chronic infections.

Early Isolates	Chronic Isolates
AMT0474-1<br>AMT0481-5<br>AMT0522-5<br>AMT0527-4<br>AMT0529-5<br>AMT0530-4<br>AMT0536-2<br>AMT0538-2<br>AMT0540-1<br>AMT0544-1<br>AMT0545-1<br>AMT0547-1<br>AMT0548-1<br>AMT0549-1<br>AMT0550-1	AMT0028-84<br>AMT0030-74<br>AMT0108-26<br>AMT0118-46<br>AMT0130-24<br>AMT0209-82<br>AMT0302-77<br>AMT0321-29<br>AMT0322-66<br>AMT0494-9

Early isolates were defined as those having been acquired from CF patients' first positive sputum culture. Chronic isolates were defined as those having been acquired from CF patients with a 4-year history of positive sputum cultures.

Supp. Table 2. Clonally-related *P. aeruginosa* isolates from CF patients.

Early Isolates	Chronic Isolates
AMT0085-2<br>AMT0085-3	AMT0085-22<br>AMT0085-23<br>AMT0085-24
AMT0086-2<br>AMT0086-3	AMT0086-24
AMT0102-2	AMT0102-38<br>AMT0102-39<br>AMT0102-40
AMT0114-2	AMT0114-47
AMT0116-2<br>AMT0116-3	AMT0116-27<br>AMT0116-29<br>AMT0116-31
AMT0159-1<br>AMT0159-2<br>AMT0159-3	AMT0159-25<br>AMT0159-26<br>AMT0159-27
AMT0166-1<br>AMT0166-2<br>AMT0166-3<br>AMT0166-4	AMT0166-23<br>AMT0166-24

Early and chronic isolates were acquired longitudinally from 7 CF patients and were collected at least 1.5 years apart. Isolates were confirmed to be clonally-related using pulsed-field gel electrophoresis.

Supp. Table 3. Primers for cytokine and reference gene targets

Gene Target	Primers
Human IL-1β	Forward: 5’-CCACAGACCTTCCAGGAGAATG-3’<br>Reverse: 5’-GTGCAGTTCAGTGATCGTACAGG-3’
Human TNF	Forward: 5’-CTCTTCTGCCTGCTGCACTTTG-3’<br>Reverse: 5’-ATGGGCTACAGGCTTGTCACTC-3’
Human IL-8	Forward: 5’-GAGAGTGATTGAGAGTGGACCAC-3’<br>Reverse: 5’-CACAACCCTCTGCACCCAGTTT-3’
Human IL-10	Forward: 5’-TCTCCGAGATGCCTTCAGCAGA-3’<br>Reverse: 5’-TCAGACAAGGCTTGGCAACCCA-3’
Human β-actin	Forward: 5’-GACTTCGAGCAAGAGATGGC-3'<br>Reverse: 5’-CACAGGACTCCATGCCCAG-3'

Supp. Table 4. Isolates of *P. aeruginosa* from the environment and from clinical, non-CF infections.

Isolate ID	Type of Isolate	Source	Location of Isolation
PR230	Environment	River Water	Woluwe, Belgium
PR231	Environment	River Water	Woluwe, Belgium
PR232	Environment	River Water	Woluwe, Belgium
PR233	Environment	River Water	Woluwe, Belgium
PR234	Environment	River Water	Woluwe, Belgium
PR235	Environment	River Water	Woluwe, Belgium
PR236	Environment	River Water	Woluwe, Belgium
PR237	Environment	River Water	Woluwe, Belgium
PR2	Clinical	Wound	Paris, France
PR3	Clinical	Wound	Paris, France
PR5	Clinical	Wound	Paris, France
PR7	Clinical	Wound	Paris, France
PR9	Clinical	Leg ulcer	Paris, France
PR80	Clinical	Urine	London, UK
PR331	Clinical	Blood	Lwiro, Congo

The type of isolate, source, and location of isolation are presented.
